# A Comparison of Standards

**Published:** 1889-05

**Authors:** 


### A COMPARISON OF STANDARDS.

From the accompaning table prepared from the annual reports of the short-term colleges of the country we are able to draw a few conclusions as to the relative standard of the several institutions therein enumerated. We have intentionally ommitted the long term colleges on the gronnd that better opportunity for instruction can be given to the same class of students in a course of two years of seven months each or in a course of three years. In making this statement, we are taking it for granted that the facilities for instruction and the instructors themselves are of equal ability and advantage respectively. We are compelled to do this in the absence of any evidence to the contrary. It is true that an effort was made to formulate a table showing the exact amount of time devoted to each subject and the number of subjects taught in each institution, but which failed to be accomplished by reason of lack of co-operation on the part of the colleges themselves.

There then remains only one basis upon which to draw a comparison of the relative standards of the various dental colleges in the country, and that is on the time-basis and the proportion of graduates turned out to the number of students matriculated. In such an estimate we must necessarily assume that students are of equal ability in all the different colleges. It will not do to credit any one institution with having a " run " of the best students as an excuse for a higher per cent. of graduates to the number of matriculates. Students average about the same. If there is any difference, those who have the least capacity will be found in institutions that have the lowest standard, by reason of the fact that they distrust themselves and seek that institution which has the reputation of giving the " easiest " examinations. We have also in this review of the question taken an average of two years so as not to deal unfairly with any : Circumstances may arise which give an institution a higher per cent. one year than another, but by taking an average of two years we avoid this liability. Yet there may be certain cases in which even this precaution will not be sufficient and we may be dealing unjustly with some of the institutions mentioned.

If such is the case, our columns are open to all and we will gladly give all an opportunity to explain. Our object is to promote thought and discussion, and not to censure any one institution.

If our premise that the students of the several institutions are of equal ability is correct, then the standard of any given institution, every-thing being equal, will be in inverse ratio to the proportion of graduates to the number of matriculants for the same year. If a two year institution sends out fifty per cent. of its matriculates each year, then matriculation in that institution is as good as a guarantee to graduation; the chance a student takes of not getting a diploma being expressed in the difference between the annual number of graduates and one-half of the matriculates.

An examination of the reports shows that the proportion in which the different colleges graduate students, varies considerably, from twenty-nine per cent. to fifty per cent. Let us put forty-three per cent. as a healthy standard, and we shall find that out of the nineteen colleges enumerated sixteen of the number fall within the standard mentioned, while three run higher. Of the six hundred and sixteen graduates for the session of '88–'89, four hundred and ninety-nine are from colleges, no one of which has passed more than forty-three per cent. of their matriculated number, while the average for these institutions is thirty-seven per cent. of the matriculation list. The other one hundred and seventeen graduates are from three institutions, in which the average per cent. of graduates is forty-eight and two-thirds per cent. of matriculates for two years.

The above should make interesting reading to the members of the State Boards of Dental Examiners, and perhaps save them some lengthy calculations. It is essential that they sooner or later take cognizance of the facts therein contained. It lies with them to regulate the standard for admission into practice and to pass on the standing of dental diplomas. In making these comparisons, we have been without bias and have presented the facts as they appear in the published reports of the several institutions. It is argued by some that our Colleges are private institutions, to be run as best suits the wishes and ideas of their board of trustees, who are generally members of the faculty also, and that they are not subject to inspection by the public.

We hold, however, to the contrary, and say that neither Dental nor medical colleges are private institutions, so long as their diplomas carry with them the license to practice. If a diploma represented the standard of education in any given institution only, and the

Condensed list of eighteen short term colleges, showing number of matriculates and graduates for 1888-9; also the per cent. of graduates to matriculates for 1888-9, the per cent. for 1888, and the average for two years. We should have been glad to make the list more complete, but were unable to get data from the other colleges, although we sent a request.

| | Matriculates. 1888-9. | Graduates. 1889. | Per cent. 1889. | Per cent. 1888. | Average for two years. |
|---|---|---|---|---|---|
| Baltimore College of Dental Surgery, Baltimore | 118 | 44 | 37 | 46 | 41 |
| Chicago College of Dental Surgery, Chicago | 154 | 64 | 41 | 34 | 37 |
| Indiana Dental College, Indianapolis | 51 | 17 | 33 | 37 | 35 |
| Kansas City Dental College, Kansas City | 30 | 11 | 36 | 35 | 35 |
| Missouri Dental College, St. Louis | 54 | 18 | 33 | 37 | 35 |
| New York College of Dentistry, New York City | 245 | 69 | 25 | 34 | 29 |
| Ohio College of Dental Surgery, Cincinnati | 150 | 65 | 43 | 29 | 36 |
| Pennsylvania College of Dental Surgery, Philadelphia | 178 | 91 | 51 | 36 | 43 |
| Philadelphia Dental College, Philadelphia | 228 | 98 | 43 | 54 | 49 |
| Columbian University, Dental Department, Washington, D. C. | 14 | 3 | 21 | * | * |
| Howard University, Dental Department, Washington, D. C. | 11 | 6 | 55 | 40 | 47 |
| University of Iowa, Dental Department, Iowa City | 79 | 21 | 26 | 40 | 38 |
| University of Maryland, Dental Department, Baltimore | 120 | 39 | 32 | 47 | 39 |
| University of Tennessee, Dental Department, Nashville | 22 | 13 | 69 | 32 | 50 |
| St. Louis C'ge of Physicians and Surgeons, Den'l Dep't, St. Louis | 14 | 6 | 44 | 39 | 41 |
| Southern Medical College, Dental Department, Atlanta, Ga. | 36 | 17 | 47 | 34 | .40 |
| School of Dentistry, Central Tennessee College, Nashville, Tenn. | 11 | 6 | 54 | 16 | 35 |
| Vanderbilt University, Department of Dentistry, Nashville, Tenn. | 95 | 28 | 29 | 42 | 35 |
| Total | 1610 | 616 | 38 | 37 | 37.5 |

* Unable to obtain figures.

license to practice was vested in and to be obtained only through a board appointed by each State for that purpose, then our colleges would not be accountable to the public in so great a measure for the way in which they manage their property. Such however is not the case.

A diploma should represent the standard of education in the institution granting it, be it scientific or literary, and no more. This rule seems to us to be more imperative in medicine and dentistry than in other profesions; with the former health and even life is at stake, yet the greatest liberty is given institutions to grant diplomas which, in many instances, carry with them the license to tamper with the public weal in a manner which is terrible to contemplate. Some decided restrictions should be thrown about the practice of those callings that have to do with the general good, and we are happy to see that such is the tendency of the times.

Let the subject be fully discussed with the view of establishing a more equitable basis upon which to judge of the character of instruction furnished. We greatly doubt whether it will ever be possible or even desirable to make the instruction alike in all institutions; it should, however, be under the control of boards appointed by the several states, and in order to give them power, the licensing power should lie with them, and not in the college which grants the diploma.

